# 

**Published:** 1886-03

**Authors:** 


Whole Number, CCCVIII. | MARCH, 1886. | Number 8. Volume ~~XXVI.~~ 25

### THE BUFFALO Medical AND Surgical Journal

ESTABLISHED IN YEAR 1844.

INDEX MEDICUS

LIBRARY INDEX S.G.C.

EDITORS:

THOS. LOTHROP, M. D. A. R. DAVIDSON, M. D.

ASSOCIATE EDITORS:

FLOYD S. CREGO, M. D. CARLTON C. FREDERICK, M. D.
FRED. R. CAMPBELL, M. D. HERBERT MICKLE, M. D.

EDWARD B. ANGELL, M. D., Rochester, N. Y.

Entered at the Post-Office at Buffalo, N. Y., as Second-class Mail Matter.

